# Morbidity and related factors among elderly people in South Korea: results from the Ansan Geriatric (AGE) cohort study

**DOI:** 10.1186/1471-2458-7-10

**Published:** 2007-01-22

**Authors:** Eun-kyung Woo, Changsu Han, Sangmee Ahn Jo, Min Kyu Park, Sungsoo Kim, Eunkyung Kim, Moon Ho Park, Juyoung Lee, Inho Jo

**Affiliations:** 1 Center for Biomedical Sciences, National Institute of Health, 194 Tongil-ro, Eunpyeong-gu, Seoul [122-701], Republic of Korea; 2 Department of Psychiatry, Korea University Medical College, 516 Gojan-1-dong, Danwon-gu, Ansan-city, Gyeonggi- do [425-707], Republic of Korea; 3 The Geriatric Health Clinic and Research Institute (GHCRI), Korea University Medical College, 516 Gojan-1-dong, Danwon-gu, Ansan-city, Gyeonggi- do [425-707], Republic of Korea; 4 Biomedical Brain Research Center, National Institute of Health, 194 Tongil-ro, Eunpyeong-gu, Seoul [122-701], Republic of Korea; 5 Department of Neurology, Korea University Medical College, 516 Gojan-1-dong, Danwon-gu, Ansan-city, Gyeonggi- do [425-707], Republic of Korea; 6 Center for Genome Research, National Institute of Health, 194 Tongil-ro, Eunpyeong-gu, Seoul [122-701], Republic of Korea

## Abstract

**Background:**

A thorough examination of the morbidity and comorbidity profiles among the elderly and an evaluation of the related factors are required to improve the delivery of health care to the elderly and to estimate the cost of that care. In South Korea where the aged population is rapidly increasing, however, to date only one study using a limited sample (84 subjects) has provided information on morbidity and related factors among the elderly. Using a large, stratified, random sample (2,767 subjects) from the population-based Ansan Geriatric study, the present study sought to assess the morbidity and comorbidity, and to determine the relationships of these variables with sociodemographic and health characteristics in elderly people in South Korea.

**Methods:**

A total of 2,767 subjects (1,215 men and 1,552 women) aged 60–84 years were randomly selected from September 2002 to August 2003 in Ansan, South Korea. Data on sociodemographic and health characteristics, and clinical diagnosis were collected using questionnaires. When available, the medical records and medications taken by the subjects were also cross-checked.

**Results:**

Of the total subjects, 78.0% reported diagnosed disease, 11.0% had been cured, and 46.8% had been diagnosed with more than two diseases. The mean number of morbidities per person among elderly Koreans was 1.62 ± 1.35 (mean ± standard deviation), and women had a greater number of diseases per person than did men. The most common morbidities were chronic diseases such as hypertension, arthritis, and diabetes mellitus. In women, osteoporosis and arthritis were the second and third most prevalent diseases, respectively. Morbidity was significantly associated with gender, employment, household income, alcohol intake, self-assessed health status, and worries about health.

**Conclusion:**

These data will enhance understanding of the patterns of health problems among elderly Koreans and will contribute to the application of appropriate intervention strategies.

## Background

The increasingly ageing populations in developed countries have recently become the focus of substantial research interest. In 2000, those aged 65 and older constituted from 6.0 to 15.5% of the populations in Asia, Europe, and North America. These figures are expected to increase to approximately 12 to 24.3% by 2030 [1]. As health status has a substantial influence on the quality of life in elderly populations, many countries have been making tremendous efforts to improve the understanding of the health status of this age group. Both perceived health and chronic illness are major elements of health status in the elderly, because perceived health declines with age and chronic health problems increase with age. Furthermore, there is a growing body of evidence indicating that older people are at risk for multiple comorbidities [2]. Therefore, a thorough examination of the morbidity and comorbidity profiles among the elderly and an evaluation of the related factors are required to improve the delivery of health care to the elderly and to estimate the cost of that care [3].

South Korea is now considered to have one of the most rapidly aging populations in the world. The percentage of those aged 65 and older was 2.9% in 1960 and rose to 7.2% in 2000. In 2030, this figure is projected to reach 24.1%, and therefore it will take only 30 years from 2000 in Korea to triple its elderly population [4]. However, to our knowledge, only one study has provided information on morbidity and related factors among the elderly in South Korea, and the sample size in that study was very limited (84 subjects) [5]. In this study, using a large, stratified, random sample (2,767 subjects) from the population-based **A**nsan **Ge**riatric (AGE) cohort study, we investigated to assess the morbidity and comorbidity among the elderly people aged 60–84 and to determine the relationships of these variables with sociodemographic and health characteristics.

## Methods

### Cohort recruitment and study population

The AGE study is an ongoing, prospective, population-based epidemiological study established in May 2002. The overall objectives of the AGE study are not only to provide basic information on the medical conditions and characteristics of the rapidly growing Korean geriatric population, but also to identify genetic and environmental risk factors of various diseases. As part of a baseline investigation to construct an elderly cohort in Korea, we sampled the elderly population living in Ansan, located in the southern part of Gyeonggi-do province, South Korea. This city comprises rural and urban area, and is now included as a metropolitan area of Seoul, a capital of South Korea, where one fourth of South Koreans live. In August 2002, 36,735 non-institutionalized civilians aged 60 to 84 years were registered as residing in Ansan [6]. On the basis of a power analysis of the sample size needed to determine the prevalence of major chronic diseases and risk factors among the elderly, a goal of interviewing 3,000 subjects was set. The sampling protocol was described previously [7,8].

Briefly, to construct the sampling framework, the telephone-subscriber data of the Korean Telecommunications Corporation, a nearly monopolistic telephone company in Korea, was first compared with residence records. Residence records were obtained from Ansan municipal office. Among 36,735 non-institutionalized civilians, a total of 19,387 records (52.8%) were matched using name and address as the key variables; the remaining 17,348 residents (47.2%) did not have a matched telephone number. To acquire a probability sample proportional to the age- and gender-specific population structure of the target population, a random sample comprising 15,392 persons (41.9%) aged 60 to 84 years was selected; 10,819 subjects were selected from the group with matched telephone numbers and 4,573 were selected from the unmatched group. A letter of invitation to participate in this study was initially sent to all 15,392 subjects. The telephone number-matched group was additionally contacted at least three times by telephone, and the unmatched group was visited at home in order to confirm their eligibility. A total of 8,999 subjects were not eligible for interview because of several 'logistic and unknown' reasons such as incorrect telephone number, incorrect address or change of address, moving, death, and not being at home for three attempted visit. One thousand nine hundred eighty nine refused our study, and therefore 4,404 were eligible for in-home or in-clinic interviews. The eligible interviewees were then stratified by gender and age (age of 60–64, 65–69, 70–74, 75–79, and 80–84) so that the total number of sample was close to 3,000 subjects that could mirror the age- and gender-specific population structure of the population of interest. Among 4,404 subjects, however, 1,637 were further excluded in this study; they were either not at home when we visited, did not appear at the clinic without prior notice, refused to participate without explanations, or ineligible for a probability sample. Therefore, a sample of 2,767 subjects was finally obtained through this method, and used for the analysis of morbidity and its related factors in this study.

Informed, written consent for participation was obtained from each individual, and the Institutional Review Board of the Ansan Hospital, Korea University Medical College approved the study protocol.

### Measures

The well-trained field survey team was recruited from among university students majoring in nursing or from among experienced interviewers. All the interviewers received two-day training to ensure uniformity. McNemar's test was used to assess the reliability between surveyors, showing no statistical difference (p > 0.05). The subjects were first asked to choose the place for interview, home or the Geriatric Health Clinic and Research Institute (GHCRI) of the Korea University Medical Center, according to their convenience. A team of two surveyors interviewed the subjects to collect all the data; approximately half (46.3%) of the subjects tested in this study were interviewed in their homes and remaining 53.7% were interviewed at clinic.

Morbidity was examined using a checklist including 35 diseases and a residual category called "other diseases" that followed the open-ended question: "Have you been diagnosed with a disease by a doctor? If so, please write down or tell all the disease names." The questionnaire also included items for cured diseases on the list of diseases. When available, the self-reported disease history was confirmed by reviewing medical records and medications, showing that the agreement between self-reports and medical records was fair to excellent (75–97%). Cured morbidities were excluded to assess the prevalence of current morbidities.

Detailed data on sociodemographic factors and personal characteristics were collected; these included age, gender, marital status, level of education, household income, employment status, religious activity, exercise, smoking, alcohol intake, self-assessed health status, and worries about health. Marital status was categorized as married (cohabiting) or single (unmarried, widowed, divorced, or separated). Smoking behavior was classified as current smoker, ex-smoker (for at least 1 year), and non-smoker, as previously described [9]. Heavy smoker was defined as a smoker who smoked at least 25 cigarettes per day [10]. Similarly, alcohol intake was classified as current drinker, ex-drinker, and non-drinker based on responses to self-administered questionnaires.

### Statistical analyses

All of the data in this study were summarized as prevalence and percentage for categorical variables. Intergroup comparisons of the sociodemographic variables and health risk factors were performed using a chi-square test for distribution and the unpaired Student *t*-test for continuous variables. The significance of various risk factors for the presence of morbidity was calculated using multiple logistic regression analysis using the SAS 9.13 (SAS Institute, Cary, NC, USA). Values of p < 0.05 were considered to be significant.

## Results

### Sociodemographic and health characteristics

Table 1 presents the distribution of the study population by each sociodemographic characteristic according to gender. More women (1,552) than men (1,215) were recruited for the study, but their distribution by age was not significantly different. The majority (80.9%) of subjects examined in this study were aged 60 to 74 years. The distribution of all of the examined sociodemographic characteristics differed significantly by gender; compared with men, women had generally lower education levels, earned lower income, were more likely to be single (unmarried, widowed, divorced, and separated), had higher unemployment levels, and participated in religious activity more regularly. Furthermore, the majority of women smoked less, consumed less alcohol, and exercised less than did men. Moreover, more women than men felt that they were unhealthy and worried about their health.

**Table 1:** The distribution of sociodemographic and health characteristics (%) in a South Korean sample population aged 60 – 84 Years, in all subjects and in men and women separately

	All subjects (N = 2767)	Men (N = 1215)	Women (N = 1552)	P*
Age (years)				
60–64	32.9	35.0	31.3	0.108
65–69	28.0	27.0	28.7	
70–74	20.0	20.5	19.7	
75–79	12.6	11.9	13.1	
80–84	6.5	5.6	7.2	
Marital status^†^				
Married	68.4	90.2	51.3	<0.001
Education (years)				
0	18.0	5.5	27.8	< 0.001
1–9	53.0	49.5	55.8	
10 ≤	29.0	45.0	16.4	
Household income (million won/month)^‡^				
< 0.5	31.7	27.4	35.1	<0.001
0.5–0.99	25.7	28.2	23.8	
1.0–1.99	27.6	28.2	27.0	
2.0 ≤	15.0	16.2	14.1	
Employment				
Yes	18.6	31.3	8.7	<0.001
Religious activity^§^				
Regularly	43.1	31.8	51.8	<0.001
Irregularly	25.1	24.2	25.9	
Never	31.8	44.0	22.3	
Exercise^∥^				
Yes	36.6	45.0	30.0	<0.001
Smoking				
Non-smoker	57.8	18.1	88.9	<0.001
Ex-smoker	24.9	49.8	5.4	
Current smoker	17.3	32.1	5.6	
Alcohol intake				
Non-drinker	53.6	21.7	78.6	<0.001
Ex-drinker	14.9	25.7	6.5	
Current drinker	31.5	52.6	14.9	
Self-assessed health status				
Good	31.7	39.8	25.3	<0.001
Moderate	20.4	24.6	17.1	
Poor	47.9	35.6	57.6	
Worries about health				
Yes	73.5	66.9	78.7	<0.001

* Comparison between men and women.

^†^Classified as married (cohabiting) or single (unmarried, widowed, divorced, or separated).

^‡ ^1 US dollar = 1055 Korea won at the time of the interviews.

^§^Religious activity data were missing for 40 subjects.

^∥^Exercise data were missing for 45 subjects.

### Morbidity profiles

Table 2 presents the distribution of morbidities diagnosed by physicians and the health characteristics of the participants. Of the total subjects, 78.0% reported at least one morbidity based on their recollection of previous diagnoses. Approximately 11.0% of the subjects reported that they had been cured of a previous condition at the date of the interview. More women (81.4%) than men (73.7%) reported that they had current disease conditions. Approximately one-third of the subjects (31.2%) were diagnosed with one current morbidity, 23.1% had more than three current morbidities, and 22.0% had no current morbidity. The mean number of morbidities per person in this sample of elderly Koreans was 1.62 ± 1.35 (mean ± standard deviation); women had a significantly higher mean number of morbidities (1.84 ± 1.44) than did men (1.33 ± 1.17).

**Table 2:** The distribution of morbidities (%) diagnosed by a physician in an elderly sample population aged 60 – 84 years, in all subjects and in men and women separately

		All subjects (N = 2767)	Men (N = 1215)	Women (N = 1552)	P*
Current morbidity status					
Yes		78.0	73.7	81.4	<0.001
No	Cured^†^	11.0	11.9	10.4	
	Never	11.0	14.4	8.2	
Number of morbidities^‡^					
0		22.0	26.3	18.6	<0.001
1		31.2	36.1	27.4	
2		23.7	21.9	25.1	
≥3		23.1	15.7	28.9	

* Comparison between men and women.

^†^Patients were told by doctors that they were recovered from the specified diseases and thus they were not currently treated for those diseases.

^‡^The mean numbers of diseases reported for all subjects, men, and women were 1.62 ± 1.35 (mean ± standard deviation), 1.33 ± 1.17, and 1.84 ± 1.44, respectively.

A total of 57 morbidities were reported in this study; the most prevalent was hypertension (37.5%), followed by arthritis (15.6%), diabetes mellitus (14.9%), osteoporosis (14.1%), and gastritis/gastric ulcer (13.1%) (Table 3). Approximately half (51.9%) of the elderly Koreans are currently having at least one cardiovascular morbidity such as hypertension, stroke, myocardial infarction, angina pectoris, and congestive heart failure. Most of the top 15 morbidities were common in both genders. Diseases of the musculoskeletal system, such as osteoporosis and arthritis, were more prevalent in women, whereas diseases of the prostate were only present among men. Other important diseases, particularly in the elderly, such as depression and insomnia, were also tested. However, the prevalence was 1.7% for depression and 1.3% for insomnia that were ranked as 16th and 21st, respectively (data not shown). We also found that the prevalence of cancer was 1.6% and ranked as the 17th in this study (data not shown).

**Table 3:** Prevalence of the most common morbidities in an elderly sample population aged 60 – 84 years, in all subjects and in men and women separately

	All subjects (N = 2767)			Men (N = 1215)			Women (N = 1552)		
	Morbidity	ICD-10* code	%	Morbidity	ICD-10* code	%	Morbidity	ICD-10* code	%

1	Hypertension	I10	37.5	Hypertension	I10	33.7	Hypertension	I10	40.5
2	Arthritis	M06	15.6	Diabetes mellitus	E14	15.6	Osteoporosis	M80-M82	24.4
3	Diabetes mellitus	E14	14.9	Gastritis/gastric ulcer	K25, K29	11.6	Arthritis	M06	23.6
4	Osteoporosis	M80-M82	14.1	Diseases of the liver	K70, K73, K74, K76	6.8	Diabetes mellitus	E14	14.4
5	Gastritis/gastric ulcer	K25, K29	13.1	Lumbar intervertebral diseases	M51, M54	6.1	Gastritis/gastric ulcer	K25, K29	14.3
6	Lumbar intervertebral diseases	M51, M54	7.3	Hyperlipidemia	E78	5.7	Lumbar intervertebral diseases	M51, M54	8.2
7	Hyperlipidemia	E78	7.0	Diseases of prostate	N40, N41, N42	5.6	Hyperlipidemia	E78	8.0
8	Diseases of the liver	K70, K73, K74, K76	5.1	Cerebrovascular diseases	I61, I63, I64, I67	5.5	Cataracts	H25, H26	5.9
9	Cataracts	H25, H26	5.1	Arthritis	M06	5.4	Cerebrovascular diseases	I61, I63, I64, I67	4.5
10	Cerebrovascular diseases	I61, I63, I64, I67	5.0	Heart diseases	I49, I50, I52	4.1	Diseases of the liver	K70, K73, K74, K76	3.9
11	Heart diseases	I49, I50, I52	3.8	Asthma	J45	4.1	Ischemic heart diseases	I20-I23	3.7
12	Ischemic heart diseases	I20-I23	3.7	Cataracts	H25, H26	4.0	Heart diseases	I49, I50, I52	3.6
13	Asthma	J45	3.3	Ischemic heart diseases	I20-I23	3.7	Asthma	J45	2.6
14	Diseases of prostate	N40, N41, N42	2.5	Allergies	L23	2.8	Disorders of thyroid gland	E00-E07	2.5
15	Allergies	L23	2.3	Respiratory diseases	J40, J44, J47	2.1	Depression	F32, F33	2.5

*International Classification of Diseases, Tenth Revision.

### Relationship of morbidity with sociodemographic and health characteristic variables

Table 4 shows the results of a multiple logistic regression model in which the presence or absence of morbidity was regressed on the sociodemographic and health characteristics. In this model, women had a significantly higher odds ratio than men (Model 1). Unemployment, lower income, and ex-drinker status were associated with higher risk of morbidity. However, the subjects who had no education or who were current smokers were more likely to have a decreased risk of morbidity compared with their counterparts. People who felt that their health status was poor or who worried about their health were likely to have significantly (~2 to 3 times) increased risk of morbidity after adjusting for sociodemographic variables (Model 2).

**Table 4:** Odds Ratios (OR) and 95% Confidence Intervals (CI) for the presence of morbidity in association with sociodemographic and health characteristics among a cohort of elderly subjects aged 60 – 84 years in Ansan, Korea

		OR	95% CI
Model 1*			
Age	(1 year)	0.99	0.98 to 1.01
Gender	Men	1.00	
	Women (ref. Men)	1.49	1.09 to 2.05
Marital status^‡^	Single	1.13	0.89 to 1.44
	Married	1.00	
Employment	Yes	1.00	
	No	1.34	1.05 to 1.71
Household income	< 0.5	1.26	0.94 to 1.68
(million won/month)^§^	0.5 – 1.99	1.47	1.08 to 1.99
	1.0 – 1.99	0.93	0.70 to 1.23
	≥ 2.0	1.00	
Education (years)	0	0.65	0.47 to 0.89
	1 – 9	0.87	0.69 to 1.09
	≥ 10	1.00	
Religious activity	Regular	1.00	
	Irregular	0.93	0.74 to 1.18
	Never	0.91	0.72 to 1.14
Exercise	Yes	1.00	
	No	0.94	0.77 to 1.15
Alcohol intake	Non-drinker	1.00	
	Ex-drinker	1.55	1.12 to 2.14
	Current drinker	0.94	0.74 to 1.20
Smoking	Non-smoker	1.00	
	Ex-smoker	1.04	0.77 to 1.41
	Current smoker	0.70	0.52 to 0.95
**Model 2**^†^			
Self-assessed health status	Good	1.00	
	Moderate	1.69	1.31 to 2.19
	Poor	2.80	2.18 to 3.59
Worries about health	Yes (ref. No)	1.92	1.55 to 2.39

* Multiple logistic analysis of sociodemographic characteristics.

^†^Multiple logistic analysis of health characteristics after adjustment for the sociodemographic characteristics.

^‡^Classified as married (cohabiting) or single (unmarried, widowed, divorced, or separated).

^§^1 US dollar = 1055 Korea won at the time of the interviews.

## Discussion

This study demonstrated that 78.0% of the elderly people aged 60 to 84 years in a South Korean sample were currently diagnosed with disease conditions and that 11.0% had been cured of at least one disease. Life style-related diseases, including hypertension, arthritis, diabetes mellitus, and osteoporosis, were the most common among these morbidities. Furthermore, unemployment, lower income, and ex-drinker status were associated with higher risk of morbidity, and women had a higher risk of morbidity than did men. Self-assessed poor health and worries about health were also significantly associated with the presence of these morbidities in elderly South Koreans.

The prevalence of morbidity in this study (78.0%) was at the lower end of the range reported in studies of four other ethnic groups [3,11-15]. Two previous studies done in the United States, the 1987 National Medical Expenditure Survey for prevalence and direct health care costs [13] and the cross-sectional study using aged Medicare beneficiaries in 1999 [14], documented that the prevalence of morbidity was 87.6% and 82.0%, respectively. Similarly, the prevalence of morbidity was 88.9% in elderly residents of Northern India [3] and 86.7% in a community-dwelling Jewish group in Israel [11], respectively. The prevalence of morbidity among non-institutionalized older people in Spain was much higher, which was 95.3% [15]. Nonetheless, direct comparisons with these six studies are not possible because of the different conditions employed; for example, this study included elderly persons aged 60–84 years, whereas the Indian and Israeli studies included subjects aged 60 years and over and those aged 75–94 years, respectively. Three other studies included the elderly aged 65 years and over.

Furthermore, the differences in the prevalence may be attributable to differences in the racial and ethnic origins of the study populations and prevailing sociodemographic differences among them. Moreover, the differences in the healthcare systems and the context of the questionnaires used to determine current disease status may also contribute to different results. Recently, the National Health and Nutrition Survey on South Korea, which was conducted in 2001, indicated that 85.4% of the elderly population over 60 years of age reported that they had experienced chronic diseases for at least the past year [16]. Clearly, this study cannot be directly compared with the 2001 study owing to differences in the inclusion criteria; the current study excluded cured morbidities to analyze the prevalence of current morbidities, whereas the 2001 study included them. Furthermore, this study used open-ended questions to collect the morbidity data, and the previous study used a pre-classified disease list. Nonetheless, if the current study had included cured morbidities in the assessment of the prevalence of morbidities, the prevalence of both cured and current morbidity (89.0%) in this study would have been comparable to those reported in the previous studies.

In this study, a total of 31.2% of the participants had one morbidity. This result is similar to previously reported data [10,12,17], although all the disease conditions differed slightly. More women (81.4%) than men (73.7%) had at least one morbidity in the South Korean study population, which is also consistent with other studies [3,14,18-20]. As elderly women were more likely than elderly men to be unemployed, and/or widowed, and to engage in less exercise, it is probable that the poorer health status of elderly women in this study may be attributable to all of these factors. Alternatively, the women may have been more interested in their own health and, consequently, may have indicated the presence of more disease conditions than did the men. Therefore, differences between men and women in the level of awareness and concern about their personal health could have affected their answers to the questions about current morbidity. In this regard, it was found that more women felt that they were unhealthy and more women worried about their health, and that these factors were associated with an increased risk of morbidity.

Approximately one third of the elderly Koreans who participated in this study reported that they had hypertension. Diabetes mellitus, gastritis/gastric ulcer, lumbar intervertebral diseases, hyperlipidemia, and cerebrovascular diseases were commonly reported morbidities among both men and women. Diseases of the musculoskeletal system, osteoporosis and arthritis, ranked as the second and the third most prevalent diseases among women, whereas diseases of the prostate were prevalent among men. The rankings of the most common chronic diseases in this study were quite similar to those from the 2001 Korean study, which showed that arthritis, hypertension, and lumbar disease were the most prevalent diseases [16]. Furthermore, this current data are also relatively consistent with those in the study of an elderly cohort in Israel [11]. In that study, the most prevalent disease was arthritis, followed by hypertension and gastrointestinal diseases. In the study from Northern India, however, anaemia and dental problems were reported to be the most prevalent diseases based on provisional diagnoses made after general physical examinations of the subjects [3]. Hypertension, chronic obstructive airway disease, cataracts, and osteoarthritis followed, and only 5% of the subjects in that study had diabetes mellitus. In the study from Spain, osteoarthritis and related diseases were found to be the most prevalent morbidities, followed by vision impairment and hypertension [15]. Again, the data in these studies are not entirely comparable owing to many differences in the conditions of the four studies.

Recently, it was reported that depression was considerably common in the elderly Koreans, with the prevalence rate of 4.6% [21]. However, the lower prevalence of depression obtained from our study cannot be directly compared with the previous study due to the difference of the study design. Target population in our study was the elderly aged 60 to 84 years, whereas the previous study was conducted on the individuals aged 65 and older. Furthermore, our data were collected largely based on what the subjects wrote down and told, whereas the previous study employed 'Diagnostic and Statistical Manual of Mental Disorders, fourth edition (DSM-IV)' for assessing depression. It was reported that lower prevalence of depression was associated with poorer self-report such as underreporting [22], and therefore the prevalence of depression from our study was likely to be underestimated. One of the possible explanations for underreporting is that many Koreans, the elderly in particular, are reluctant to expose their mental health problems because there is a social prejudice for the mental condition. Further studies are necessary to clarify this issue. In this study, cancer failed to be among the most 15 prevalent current diseases, but ranked as the third most common cured morbidity (data not shown). This is probably attributable to the recovery or death of the subjects with cancer so that a relatively small number of current cancer patients were included in this study.

Using multiple regression analysis, it was found that progressive ageing was not significantly related to morbidity among the elderly, although old age is usually accompanied by a decline in physical fitness and increasing experience of body aches and pains [3]. Therefore, it was concluded that most of the major chronic diseases in the current sample were established or diagnosed before the age of 60 and that thereafter a large proportion of the subjects had chronic diseases. Alternatively, this result may imply the underreporting from the older age group among our study sample, since they are most likely to have more chronic diseases than younger group. Older age was known to be associated with poorer health literacy [23], which might lead to failure of adequate reporting of disease status. In this study, we found that lower level of education was associated with decreased risk of morbidity. This result can also be explained by the underreporting. It was informed that the lower level of education, the poorer health literacy [23]. Although ex-drinkers were more likely to have a higher risk of chronic diseases compared with non-drinkers, this cannot be interpreted as meaning that a history of alcohol intake contributes to increased risk of chronic disease because the setting of the present study was cross-sectional. An alternative interpretation is that the ex-drinkers stopped consuming alcohol because of the onset of a disease. Similarly, this study also shows that current smokers were likely to have less risk of morbidity compared with non-smokers. This result is interesting because smoking has known to be associated with increased risk of chronic diseases. In this study, however, only 3.6% of current smokers were heavy smokers (data not shown), and therefore it would also be reasonable to interpret this finding as indicating that elderly people with fewer disease conditions would be more likely to smoke or continue smoking.

After adjusting for sociodemographic variables, chronic disease was strongly associated with self-assessed health status and worries about health. Multiple studies had demonstrated that self-assessed health status is a good predictor of mortality and functional ability, even after controlling for objective measurements of medical morbidity (for example, laboratory tests or physician reports) [24,25]. In this regard, it was reported that self-assessed health status is a multifaceted, nuanced indicator of underlying health status that incorporates different dimensions of health (including physical disability and functional limitations), severity of any conditions, and comorbidity [19]. In this study, even though this factor is a subjective classification, the group that self-assessed their health status as "poor" had a higher odds ratio for morbidity than did the group with "moderate" self-assessed health status. These findings support the possibility that self-assessed health status is a good indicator for morbidity.

The present study has several limitations that must be addressed in future studies. First, because the sample was drawn from one limited geographic area within Korea, the results cannot properly be generalized to the national population. Recently, Korea changes so rapidly and therefore many rural districts are quickly urbanizing. Ansan is one good example among many urbanized cities in Korea. Furthermore, because of data obtained in this study are consistent with previous study in Korea (see discussion), their validity is probable. Second, because of the cross-sectional design, this study was unable to determine whether the various factors that showed correlations with morbidity in the elderly are antecedent to or consequences of morbidity. To resolve this question, a longitudinal follow-up study using this cohort is currently being conducted. Third, geriatric epidemiologists are concerned that misreporting on self-assessments may increase with age and may vary greatly depending on the disease considered. Moreover, because morbidity was investigated using a questionnaire and the subjects were instructed to answer 'yes' only for diagnosed diseases, any unexpressed or undiagnosed diseases were not investigated. Nevertheless, the present study also has an advantage, which was based on a large, stratified, random sample of the elderly in Korea.

## Conclusion

Our data can be used to assess the patterns of health problems among the elderly in South Korea. Furthermore, this study identifies an urgent need for nationwide efforts to develop various intervention programs for decreasing age-associated morbidity or comorbidity. Similar intervention programs can be also applicable to other developing countries where aging populations rapidly increase.

## Competing interests

The author(s) declare that they have no competing interests.

## Authors' contributions

EW and CH: co-first authors. They contributed to analysis and interpretation of data, and preparation of manuscript. SAJ: co-corresponding author. She was involved in coordinating AGE study and contributed to study design, analysis and interpretation of data, and preparation of manuscript. She also contributed to financial supports for this study. MKP: deceased. He was a principal investigator at the early phase of this study. SK, EK, MP and JL: data managers. They were involved in the study design and responsible for data collection and analysis. IJ: corresponding author. He was responsible for all the process of this study. All authors read and approved the final manuscript.

## Pre-publication history

The pre-publication history for this paper can be accessed here:


